# RRS1 Promotes Retinoblastoma Cell Proliferation and Invasion via Activating the AKT/mTOR Signaling Pathway

**DOI:** 10.1155/2020/2420437

**Published:** 2020-11-02

**Authors:** Xuejing Yan, Shen Wu, Qian Liu, Jingxue Zhang

**Affiliations:** ^1^Beijing Institute of Ophthalmology, Beijing Tongren Eye Center, Beijing Tongren Hospital, Capital Medical University, Beijing, China; ^2^Beijing Ophthalmology & Visual Sciences Key Laboratory, Beijing, China

## Abstract

Ribosome biogenesis regulatory protein homolog (RRS1) is a protein required for ribosome biogenesis. Recent studies have identified an oncogenic role of RRS1 in some cancers, whereas the involvement of RRS1 in retinoblastoma (RB) remains to be determined. In this study, we aimed to explore the role of RRS1 in RB. We found that the expression of RRS1 was increased in RB tissues and cells. Lentivirus-mediated RRS1 overexpression promoted the proliferation, growth, and invasion of RB cells. Opposite results were found in RRS1 knockdown cells. In addition, RRS1 silencing induced cell cycle arrest at the G1 phase and apoptosis in RB cells, while RRS1 ectopic expression exhibited the opposite effect. At the molecular level, RRS1 activated the AKT/mTOR signaling pathway, inhibition of which largely blunted the proliferation, growth, and invasion of RB cells. Our study suggests that RRS1 functions as an oncogene in RB through activating the AKT/mTOR signaling pathway.

## 1. Introduction

Retinoblastoma (RB) is the most common intraocular malignancy among infants and children younger than 5 years throughout the world [1, 2]. Until now, routine therapies for RB patients have included chemotherapy, radiotherapy, laser photocoagulation, thermotherapy, cryotherapy, and enucleation [3], but the treatment outcomes are far from satisfactory, and the relapse rate remains high. RB was first identified as being caused by inactivating mutations of the tumor suppressor gene RB1 [4]. However, some patients do not harbor an RB1 mutation. Furthermore, the development of RB is often a complex and multistep process that is correlated with various genetic or epigenetic changes, even within one patient. Therefore, understanding the molecular mechanisms responsible for the growth and migration of RB may help develop novel therapeutic targets and effective treatment strategies for this deadly disease.

Ribosome biogenesis regulatory 1 homolog (RRS1) protein is conservatively expressed in eukaryotes [5]. Recent studies have shown that RRS1 interacts with RPF2 to form a complex that regulates the maturation of the 60S ribosomal subunit [6]. In this way, it plays an important role in ribosome biogenesis [7]. Since ribosome biogenesis is a critical event during protein synthesis, researchers have paid attention to the pathological role of RRS1 in cancer development. RRS1 is highly expressed in colorectal cancer (CRC) tissues, and its expression is inversely correlated with the survival of CRC patients. Knockdown of RRS1 suppresses the proliferation and growth of CRC cells [8]. Likewise, silencing of RRS1induces the apoptosis of hepatocellular carcinoma (HCC) cells, resulting in reduced HCC cell viability [9]. Furthermore, the oncogenic role of RRS1 has also been identified in breast cancer [10, 11]. However, little is known about the involvement of RRS1 in RB.

In this study, we aimed to investigate the role of RRS1 in RB. We found that RRS1 was overexpressed in RB tissues and cells. In vitro experiments demonstrated that RRS1 promoted the proliferation, growth, and invasion of RB cells. The cell cycle and apoptosis were regulated by RRS1. At the molecular level, the AKT/mTOR signaling pathway was activated by RRS1. AKT inhibitor suppressed the proliferation and invasion of RRS1 overexpressing RB cells. We suggest that RRS1 functions as an oncogene in RB via activating the AKT/mTOR signaling pathway.

## 2. Materials and Methods

### 2.1. Microarray of Human RB Tissues

The BC35111a microarrays of human RB tissues and adjacent normal samples were obtained from Xi'an Alena Biotechnology Ltd., Co. (Shanxi, China). This array contained a total of 14 samples with 6 stage I, 6 stage II, 1 stage III, and 1 stage IV RB tissue samples and 6 adjacent normal samples. A core represented a separate case, and each sample was fixed in formalin. Five-micrometer-thick slices coated with paraffin were subjected to immunohistochemical staining of RRS1.

### 2.2. Immunohistochemistry (IHC) Analysis of RRS1

The microarrays were deparaffinized in xylene and hydrated in graded alcohol and underwent antigen retrieval, and endogenous peroxidase was blocked by a citrate buffer (pH = 6) and 3% hydrogen peroxide. After blocking with 10% serum, the sections were incubated with anti-RRS1 (Abcam, 1 : 200) primary antibody at 4°C overnight, followed by incubation with the secondary antibodies. DAB was used as the chromogen. The images were photographed under a microscope.

### 2.3. Cell Culture

The human retinal pigment epithelial cell line ARPE-19 was kindly provided by Doctor Zengyi Wang (Beijing Tongren Hospital), and the RB cell lines Y79 and WERI-Rb-1 were purchased from the American Type Culture Collection (Manassas, VA, USA) and Shanghai Cell Bank, Chinese Academy of Sciences, respectively. The cells were cultured in RPMI-1640 medium (HyClone, USA) supplemented with 20% fetal bovine serum (FBS) and 1% penicillin/streptomycin solution (Gibco, USA), and cells were maintained at 37°C with 5% CO_2_.

### 2.4. Lentivirus-Mediated RRS1 Knockdown

pLL3.7 lentivirus vectors were used for RRS1 knockdown. In brief, pLL3.7 vectors (shCtrl, shRRS1-1, or shRRS1-2) and packaging vectors (REV, VSVG, and pMDL) were cotransfected into 293FT cells. Forty-eight hours later, the virus supernatants were collected and filtered through a 0.45 *μ*m filter. The supernatants were stored at -80°C until use. Upon reaching a density of 30%-50%, Y79 or WERI-Rb-1 cells were infected with shCtrl, shRRS1-1, or shRRS1-2 lentiviruses with polybrene. qRT-PCR and western blotting were performed to detect the knockdown efficacy. The shRNA sequences were as follows: RRS1-1 forward: 5′-tgggccaccaataagcagatgattcaagagatcatctgcttattggtggcccttttttc-3′, RRS1-1 reverse: 5′-tcgagaaaaaagggccaccaataagcagatgatctcttgaatcatctgcttattggtggccca-3′. RRS1-2 forward: 5′-tgcaactgctcatcaaccagctttcaagagaagctggttgatgagcagttgcttttttc-3′, RRS1-2 reverse: 5′-tcgagaaaaaagcaactgctcatcaaccagcttctcttgaaagctggttgatgagcagttgca-3′.

### 2.5. RRS1 Overexpression Assay

The pCDH lentivirus vector was used for RRS1 overexpression. Briefly, the RRS1 coding sequence (CDS) was cloned into pCDH. Empty vectors or pCDH-RRS1 vectors were cotransfected with the packaging vectors PSPAX2 and PDM2G into 293FT cells. Two days after transfection, the virus supernatants were filtered with 0.45 *μ*m filters and used for the infection of Y79 or WERI-Rb-1 cells.

### 2.6. Quantitative Real-Time PCR (qRT-PCR)

The cells were harvested using TRIzol reagent (Invitrogen), and the RNA was extracted according to the instructions. One microgram of total RNA was reversely transcribed with the ReverTra Ace® qPCR RT Master Mix (TOYOBO, Japan). cDNA quantification was analyzed with the TransStart Top Green qPCR SuperMix (TransGen Biotech) on a Bio-Rad machine. The primer sequences were as follows: RRS1 forward, 5′-GCGTGGAAGAGGCGATAGTG-3′ and reverse, 5′-CTGCTGCCAGCGTGTAAGTG-3′; GAPDH forward, 5′-TGACTTCAACAGCGACACCCA-3′ and reverse, 5′-CACCCTGTTGCTGTAGCCAAA-3′. GAPDH served as the internal control.

### 2.7. Western Blot

The proteins from ARPE-19, Y79, and WERI-Rb-1 cells were extracted using RIPA buffer (Beyotime). The concentration was determined by a BCA kit. Equal amounts of the total proteins were separated by sodium dodecyl sulfate-polyacrylamide gel electrophoresis (SDS-PAGE) and transferred to PVDF membranes. After blocking with 5% nonfat milk for 1 hour, the membranes were incubated with primary antibodies at 4°C overnight and with secondary antibodies at room temperature for 2 hours. Then, the membranes were washed with TBST and subjected to chemiluminescence analysis using the ECL-Plus kit (Amersham Biosciences). The antibody against RRS1 was from Abcam. Antibodies against p-AKT (1 : 1000), AKT (1 : 1000), p-mTOR (1 : 1000), and mTOR (1 : 1000) were from Cell Signaling Technology. GAPDH primary antibody (1 : 10000) and all the secondary antibodies (1 : 10000) were from Proteintech.

### 2.8. Cell Proliferation

CCK8 assay was performed to detect cell proliferation. Y79 and WERI-Rb-1 cells were seeded into 96-well plates at a density of 2000 cells/well. Then, 1, 2, 3, 4, and 5 days later, 20 *μ*l of CCK8 solution was added to each well. The plates were maintained at 37°C for 2 hours. Then, spectrometric absorbance at 450 nm was detected on the microplate reader.

### 2.9. Methylcellulose Colony Formation Assay

The methylcellulose colony formation assay was performed to examine cell growth. Briefly, a total of 3 ml of culture, which contained 0.9 ml of RB cells (1∗10^3^ cells/ml), 1.2 ml of 2.1% (*w*/*v*) methylcellulose, and 0.9 ml of FBS, was seeded on 35 mm plates with gridlines. Ten days later, the plates were photographed and the colonies were counted.

### 2.10. Invasion Assay

The invasion capacity of RB cells was measured by Transwell assay. The cells were seeded on the upper chamber of the Transwell unit containing a Matrigel-coated filter. Three days later, the RB cells invaded through the filters to the lower chamber. The number of viable cells was examined by trypan blue staining in which only the unstained cells were counted.

### 2.11. Cell Apoptosis

Cell apoptosis was measured by using PI/Annexin V-APC staining and analyzed on flow cytometry. Briefly, cells were harvested and washed by PBS. After staining with PI and Annexin V, cell apoptosis was detected on flow cytometry.

### 2.12. Cell Cycle

Cell cycle was measured by using PI staining and analyzed on flow cytometry. Briefly, cells were washed by iced PBS for three times and fixed in 70% ethanol overnight. After staining with PI, cell cycle was detected on flow cytometry.

### 2.13. Statistical Analysis

All the statistical data are shown as the mean ± standard error of the mean (SEM) of at least three independent repeats. Statistical analysis was performed by GraphPad Prism software. Student's *t* test was used to analyze the difference between two groups. One-way ANOVA was used when there were more than two groups. A *p* value of less than 0.05 was considered significant.

## 3. Results

### 3.1. RRS1 Is Upregulated in RB Tissues and Cells

**Figure 1:**
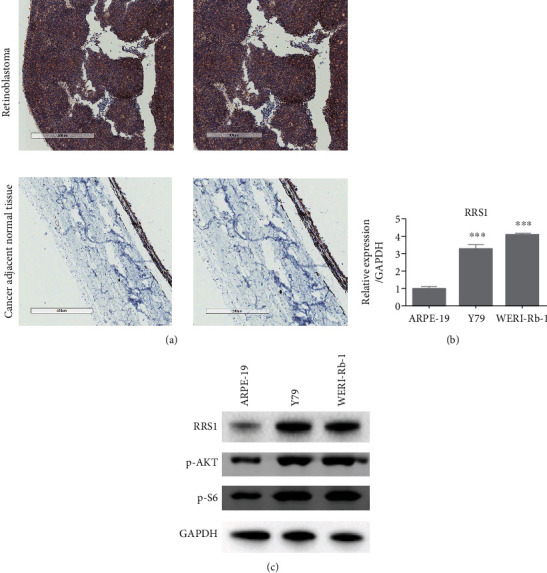
RRS1 is upregulated in RB tissues. (a) IHC staining of RRS1 in retinoblastoma tissues and adjacent normal tissues. (b) qRT-PCR analysis of RRS1 in the human retinal pigment epithelial cell line ARPE-19 and in the RB cell lines Y79 and WERI-Rb-1. ^∗∗∗^*p* < 0.001. (c) Western blot analysis of RRS1, p-AKT, and p-S6 in the human retinal pigment epithelial cell line ARPE-19 and in the RB cell lines Y79 and WERI-Rb-1.

We first investigated the clinical relevance of RRS1 in RB using IHC experiments. The results showed that RRS1 was significantly upregulated in RB tissues compared with the adjacent normal tissue (Figure 1(a) and Table 1). Furthermore, the abundance of RRS1 was detected in the Y79 and WERI-Rb-1 RB cells and in the ARPE-19 human retinal pigment epithelial cells. qRT-PCR analysis showed that RRS1 mRNA levels were higher in Y79 and WERI-Rb-1 cells than in ARPE-19 (Figure 1(b)). Consistent results for the protein level were observed in these cells (Figure 1(c)). In addition, the phosphorylation of AKT and S6 was enhanced in Y79 and WERI-Rb-1 cells (Figure 1(c)). These results suggest that RRS1 may participate in the pathogenesis of RB.

### 3.2. RRS1 Promotes the Proliferation and Growth of RB Cells

**Figure 2:**
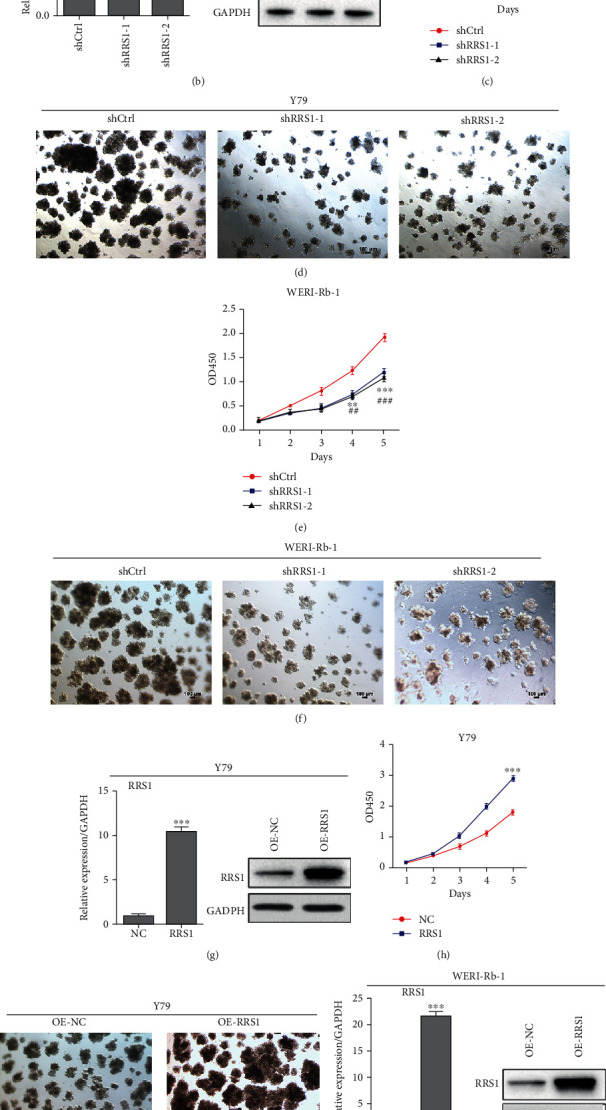
RRS1 promotes the proliferation and growth of RB cells. (a) Y79 cells transfected with shCtrl, shRRS1-1, and shRRS1-2 lentiviruses were subjected to qRT-PCR and western blot analysis of RRS1. ^∗∗∗^*p* < 0.001. (b) WERI-Rb-1 cells transfected with shCtrl, shRRS1-1, and shRRS1-2 lentiviruses were subjected to qRT-PCR and western blot analysis of RRS1. ^∗∗∗^*p* < 0.001. (c) Cells described in (a) were subjected to CCK8 assay. ^∗∗,##^*p* < 0.01, ^∗∗∗,###^*p* < 0.001. (d) Cells described in (a) were subjected to colony formation analysis. (e) Cells described in (b) were subjected to CCK8 assay. ^∗∗,##^*p* < 0.01, ^∗∗∗,###^*p* < 0.001. (f) Cells described in (b) were subjected to colony formation analysis. (g) Y79 cells transfected with Ctrl and RRS1 overexpression lentivirus were subjected to qRT-PCR and western blot analysis of RRS1. ^∗∗∗^*p* < 0.001. (h) Y79 cells transfected with Ctrl and RRS1 overexpression lentivirus were subjected to CCK8 assay. ^∗∗^*p* < 0.01, ^∗∗∗^*p* < 0.001. (i) Y79 cells transfected with Ctrl and RRS1 overexpression lentiviruses were subjected to colony formation analysis. (j) WERI-Rb-1 cells transfected with Ctrl and RRS1 overexpression lentiviruses were subjected to qRT-PCR and western blot analysis of RRS1. ^∗∗∗^*p* < 0.001. (k) WERI-Rb-1 cells transfected with Ctrl and RRS1 overexpression lentiviruses were subjected to CCK8 assay. ^∗∗^*p* < 0.01, ^∗∗∗^*p* < 0.001. (l) WERI-Rb-1 cells transfected with Ctrl and RRS1 overexpression lentiviruses were subjected to colony formation analysis.

Next, we explored the role of RRS1 in RB cell proliferation and growth. Lentivirus-mediated knockdown assays were performed in Y79 and WERI-Rb-1 cells. qRT-PCR and western blot results showed that RRS1 was efficiently silenced in both cells (Figures 2(a) and 2(b)). Based on the CCK8 and colony growth assays, we found that RRS1 knockdown led to suppressed proliferation and growth of Y79 (Figures 2(c) and 2(d)) and WERI-Rb-1 cells (Figures 2(e) and 2(f)). To validate our findings, RRS1 was overexpressed in Y79 and WERI-Rb-1 cells, and the viability was measured in both cells. qRT-PCR and western blot results indicated that RRS1 was obviously overexpressed in these cells (Figures 2(g) and 2(j)). As expected, ectopic expression of RRS1 promoted the proliferation and growth of Y79 (Figures 2(h) and 2(i)) and WERI-Rb-1 cells (Figures 2(k) and 2(l)). Taken together, RRS1 is a potential oncogene in RB.

### 3.3. RRS1 Regulates the Cell Cycle, Apoptosis, and Invasion in RB Cells

**Figure 3:**
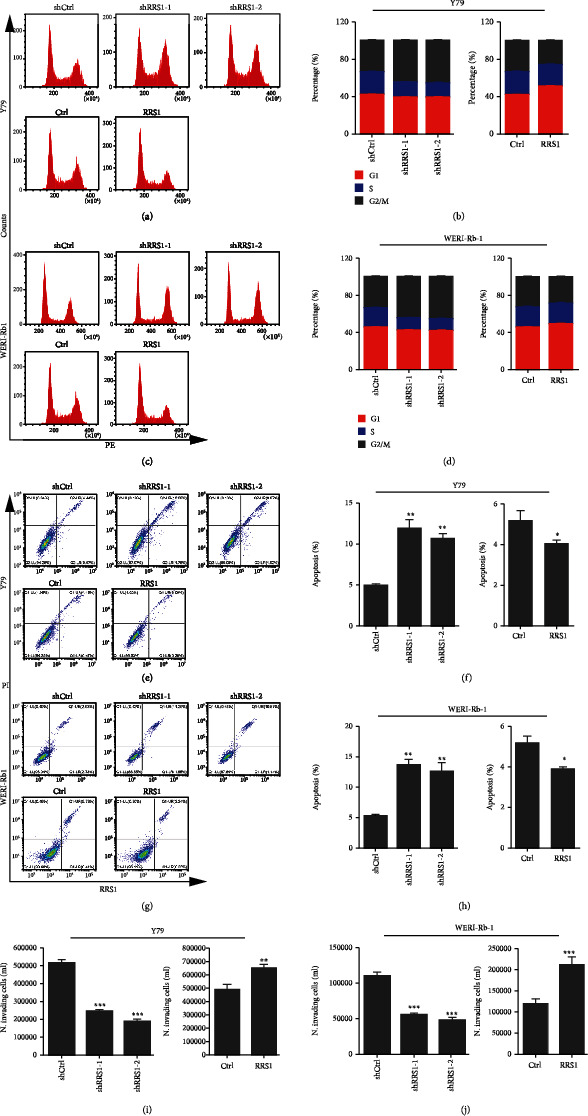
RRS1 regulates the cell cycle, apoptosis, and invasion of RB cells. (a, b) RRS1 knockdown and overexpressing Y79 cells and the respective controls were subjected to flow cytometry analysis of cell cycle distribution. (a) FCS images of cell cycle. (b) Quantification of cell cycle distribution. (c, d) RRS1 knockdown and overexpressing WERI-Rb-1 cells and the respective controls were subjected to flow cytometry analysis of cell cycle distribution. (c) FCS images of cell cycle. (d) Quantification of cell cycle distribution. (e, f) RRS1 knockdown and overexpressing Y79 cells and the respective controls were subjected to apoptosis analysis. (e) FCS images of cell apoptosis. (f) Quantification of cell apoptosis. ^∗^*p* < 0.05, ^∗∗^*p* < 0.01. (g, h) RRS1 knockdown and overexpressing WERI-Rb-1 cells and the respective controls were subjected to apoptosis analysis. (g) FCS images of cell apoptosis. (h) Quantification of cell apoptosis. ^∗^*p* < 0.05, ^∗∗^*p* < 0.01. (i) Transwell analysis of invaded Y79 cells transfected with shCtrl, shRRS1-1, and shRRS1-2 or with control and RRS1 overexpression lentiviruses. ^∗∗^*p* < 0.01, ^∗∗∗^*p* < 0.001. (j) Transwell analysis of invaded WERI-Rb-1 cells transfected with shCtrl, shRRS1-1, and shRRS1-2 or with control and RRS1 overexpression lentiviruses. ^∗∗∗^*p* < 0.001.

Previous studies reported that RRS1 knockdown induced cell cycle arrest at the G2/M phase and apoptosis in colorectal cancer cells [8]. Therefore, we investigated whether RRS1 overexpression in RB was associated with cell cycle progression and apoptosis. To address this question, RRS1 was silenced or overexpressed and the RB cells were subjected to cell cycle distribution analysis on a flow cytometer. We found that RRS1 knockdown led to a decreased S phase and increased G2/M phase distribution in Y79 and WERI-Rb-1 cells. In contrast, reduced G2/M phase distribution was observed in RRS1 overexpressing Y79 and WERI-Rb-1 cells (Figures 3(a)–3(d)). In addition, RRS1 negatively regulated apoptosis in both cell types (Figures 3(e) and 3(h)). We also analyzed the role of RRS1 in RB cell invasion. The results showed that downregulation of RRS1 suppressed the invasion of RB cells, while RRS1 upregulation promoted RB cell invasion (Figures 3(i) and 3(j)). Collectively, RRS1 participates in the cell cycle, apoptosis, and invasion functions in RB cells.

### 3.4. RRS1 Activates the AKT/mTOR Signaling Pathway

**Figure 4:**
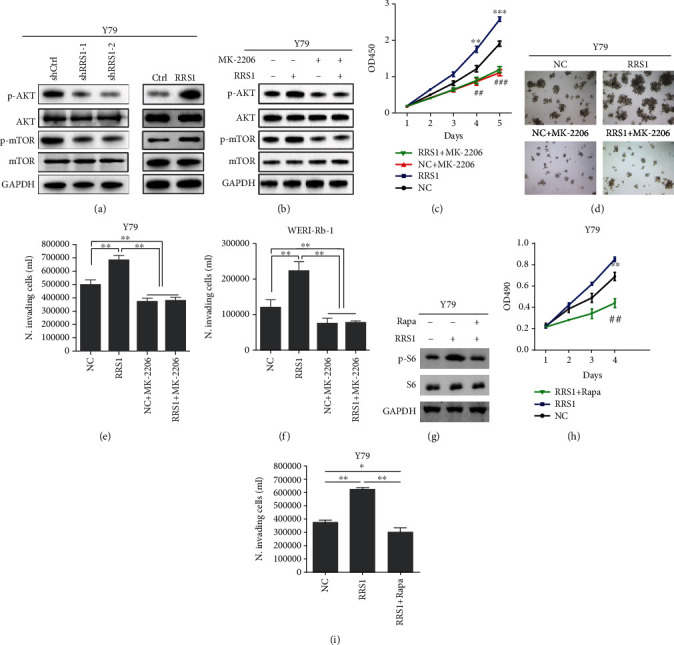
RRS1 activates the AKT/mTOR signaling pathway. (a) Western blot analysis of AKT and mTOR phosphorylation in RRS1 knockdown and the control and in RRS1 overexpressing and the control Y79 cells. (b) Control and RRS1 overexpressing Y79 cells treated with or without MK-2206 were subjected to western blot analysis with the indicated antibodies. (c) Control and RRS1 overexpressing Y79 cells were treated with or without MK-2206, and the cell viability was analyzed by CCK8 assay. ^∗∗,##^*p* < 0.01, ^∗∗∗,###^*p* < 0.001. (d) Control and RRS1 overexpressing Y79 cells were treated with or without MK-2206 and then subjected to colony formation analysis. (e) Control and RRS1 overexpressing Y79 cells were treated with or without MK-2206 and then subjected to Transwell analysis of invaded cells. ^∗∗^*p* < 0.01. (f) Control and RRS1 overexpressing WERI-Rb-1 cells were treated with or without MK-2206 and then subjected to Transwell analysis of invaded cells. ^∗∗^*p* < 0.01. (g) Control and RRS1 overexpressing Y79 cells and RRS1 overexpressing Y79 cells treated with rapamycin were subjected to western blot analysis with the indicated antibodies. (h, i) Control and RRS1 overexpressing Y79 cells and RRS1 overexpressing Y79 cells treated with rapamycin were subjected to CCK8 analysis of cell proliferation (h) and Transwell analysis of invaded cells (i). ^∗^*p* < 0.05, ^∗∗,##^*p* < 0.01.

The AKT/mTOR signaling pathway is commonly activated in different cancers, including RB. Western blot results showed that RRS1 knockdown reduced the phosphorylation but not the protein abundance of AKT and mTOR (Figure 4(a)). Consistently, ectopic expression of RRS1 upregulated the phosphorylated level of AKT and mTOR (Figure 4(a)). Then, we treated control and RRS1 overexpressing RB cells with or without the AKT inhibitor MK-2206. MK-2206 obviously suppressed the activity of AKT and mTOR (Figure 4(b)). Furthermore, MK-2206 exhibited a larger inhibitory effect on the proliferation and growth of Y79 cells with ectopic RRS1 expression (Figures 4(c) and 4(d)). MK-2206 also significantly inhibited the invasion of RRS1 overexpressed Y79 and WERI-Rb-1 cells, while had minimal effect on the invasion of NC cells (Figures 4(e) and 4(f)). Lastly, we treated RRS1 overexpressed cells with rapamycin, a specific inhibitor of mTOR activity. Phosphorylation of S6, an indicator of mTOR activity, was increased in RRS1 overexpressed cells, which was significantly reduced after rapamycin treatment (Figure 4(g)). Rapamycin treatment suppressed the proliferation and invasion of Y79 cells (Figures 4(h) and 4(i)). Our results indicate that RRS1 promotes RB growth through activating the AKT/mTOR signaling pathway.

## 4. Discussion

Retinoblastoma usually causes loss of vision and threatens the life of human beings. Even though a large amount of studies has identified critical tumor suppressors or oncogenes that trigger this malignancy, including RB1, PTEN, and MYC [12–14], effective targeted therapies remain far from being clinically satisfactory. In this study, we focused on the role of RRS1 in promoting the proliferation and invasion of RB, as RRS1 was significantly upregulated in RB tissues and cells. We also found that RRS1 enhanced AKT/mTOR signaling activity, which is frequently activated in various cancers.

RRS1 was primarily found as a regulator responsible for ribosome biogenesis in Saccharomyces cerevisiae [5]. Basically, structural and functional studies suggest that RRS1 interacts with RPF2 to modulate ribosome biogenesis [6, 15]. This interaction also plays an important role in forming an authentic immune complex in tobacco and in Arabidopsis [16]. As tumor cells often require a large amount of protein synthesis, the role of RRS1 in cancer development has been investigated. It has been shown that RRS1 is highly expressed in HCC, breast cancer, papillary thyroid carcinoma, and CRC [8–11, 17]. Here, we found that RRS1 was upregulated in RB tissues compared with the adjacent normal tissues. Using lentivirus-mediated knockdown and overexpression, we demonstrated that RRS1 promoted the proliferation, growth, and invasion of RB cells. Recently, a study showed that during interphase, RRS1 is located at the nucleolus and contributes to chromosome condensation [18]. In addition, knockdown of RRS1 negatively regulates the cell cycle progression and induces apoptosis in cancer cells [8]. Our results suggested that RRS1 knockdown induced cell cycle arrest at the G2/M phase and induced apoptosis in RB cells. Opposite results were observed in RRS1 overexpressing cells. The major reason that RB causes mortality is metastasis in the patients whose tumors show resistance to routine therapies. Based on our results, we found that RRS1 also promoted the invasion of RB cells. Therefore, RRS1 supports the proliferation, growth, and invasion of RB.

Due to the dysregulation of upstream tumor suppressors or oncogenes, such as PTEN, TSC1/2, PI3K, or IRS1, AKT/mTOR signaling is frequently activated in various cancers [19–21]. This pathway plays an important role in various cell processes, including proliferation, survival, and metabolism [22]. It is well known that rapamycin, the inhibitor of mTOR, has been widely used in the clinic for tumor treatment. Understanding the molecular triggers of this pathway may help with the clinical use of rapamycin for RB. Our previous study demonstrated that microRNA-448 inhibited RB growth by negatively regulating ROCK1 and the AKT/mTOR cascade [23]. Another study showed that the c-Met/AKT/mTOR pathway was suppressed by miR-140-5p in RB. Nevertheless, the upstream factors that activate AKT/mTOR in RB remain largely unknown. Here, we showed that RRS1 knockdown inhibited the phosphorylation but not the total protein expression of AKT and mTOR. Consistently, RRS1 overexpression led to activation of both AKT and mTOR. Importantly, suppression of AKT activity by MK-2206 obviously retarded the proliferation, growth, and invasion of RB cells. Our results suggest that targeting this signaling pathway may be effective in RB with overexpression of RRS1.

In summary, we provided evidence that RRS1 is a protooncogene in RB. Upregulation of RRS1 in RB promoted its growth and invasion. The frequently activated AKT/mTOR signaling in tumors was triggered by RRS1 in RB. Inhibition of AKT blunted the viability of RRS1 overexpressing RB cells. Our findings highlight the role of RRS1 in RB and underscore the downstream targets of RRS1.

## Figures and Tables

**Table 1:** The expression of RRS1 in human retinoblastoma and normal tissues.

	No.	RRS1-positive	RRS1-negative	*χ* ^2^	*p*
Retinoblastoma	14	11 (78.6%)	3 (21.4%)	6.222	0.013
Cancer adjacent normal tissue	6	1 (16.7%)	5 (83.3%)		

## Data Availability

The data used to support the findings of this study are available from the corresponding author upon request.
